# A Prospective Study of Reverse Axillary Mapping During Modified Radical Mastectomy and Its Association With Postoperative Lymphedema

**DOI:** 10.7759/cureus.117655

**Published:** 2026-10-08

**Authors:** Kishan Atchutuni, Vivian Anandith Paul

**Affiliations:** 1 Surgical Oncology, MNJ Institute of Oncology and Regional Cancer Centre, Hyderabad, IND

**Keywords:** axillary lymph node dissection, breast cancer, lymphedema, modified radical mastectomy, reverse axillary mapping

## Abstract

Background and objective

During axillary lymph node dissection (ALND), an axillary reverse mapping (ARM) technique was developed to identify and safeguard upper extremity lymphatic drainage channels. Preservation of these lymphatic structures may reduce the risk of breast cancer-related lymphedema while maintaining adequate oncological clearance of the axilla. This study aimed to evaluate postoperative and oncological outcomes associated with preserving the ARM during modified radical mastectomy (MRM).

Methodology

This prospective comparative study included 100 women with breast cancer undergoing MRM with ALND. Patients were equally divided into two groups of 50 patients each. In Group A, ARM-identified lymphatic structures outside the axillary dissection field were preserved, whereas in Group B, ARM lymph nodes and lymphatic structures encountered within the dissection field were sacrificed. Demographic characteristics, total lymph node yield, ARM lymph node yield, metastatic ARM-stained lymph nodes, postoperative lymphedema, lymphocele formation, drain output, and drain-removal time were compared between the groups. Continuous variables were expressed as means ± standard deviations (SDs), whereas categorical variables were expressed as frequencies and percentages. Statistical significance was defined as a two-sided p-value of less than 0.05.

Results

Group A's mean age was 50.08 ± 11.866 years, whereas Group B's was 51.28 ± 12.584 years. There was no statistically significant difference between the two groups (p = 0.625). Additionally, the distribution of surgical laterality was similar (p = 0.227). There was no significant difference between the mean total lymph node yield of 9.84 ± 3.177 in Group A and 10.96 ± 3.398 in Group B (p = 0.092). ARM lymph node yield was significantly higher in Group B than in Group A (1.38 ± 0.53 vs. 0 ± 0; p = 0.001). Metastatic ARM-stained lymph nodes were uncommon and did not differ significantly between groups (p = 0.155). Six patients (12.0%) in Group A and nine patients (18.0%) in Group B experienced postoperative lymphedema (p = 0.401). Lymphocele occurred in one (2.0%) patient in Group A and three (6.0%) patients in Group B (p = 0.307). Mean drain output was 337 ± 183.181 in Group A and 353 ± 199.055 in Group B (p = 0.676), while drain-removal time did not differ significantly between groups (p = 0.070).

Conclusions

ARM preservation during MRM with ALND was feasible and was not associated with a significant reduction in total axillary lymph node yield. The ARM-preservation group had a numerically lower incidence of postoperative lymphedema, but this difference was not statistically significant. The low frequency of metastatic ARM-stained lymph nodes supports selective preservation in appropriately selected patients. In contrast, suspicious or oncologically involved ARM nodes should be excised to maintain oncological safety. Larger prospective studies with longer follow-up and standardized lymphedema assessment are warranted.

## Introduction

The main purposes of axillary lymph node dissection (ALND) in patients with breast cancer are axillary staging and guiding adjuvant treatment choices [1-3]. It also contributes to local disease control within the axilla. However, ALND is associated with several postoperative complications, of which upper-extremity lymphedema remains a significant and potentially debilitating morbidity among breast cancer survivors.

Despite advances aimed at reducing the morbidity associated with axillary surgery, lymphedema remains a considerable postoperative concern. The reported incidence of lymphedema ranges from 11.8% to 53.5% following ALND and from 0% to 15.8% following sentinel lymph node biopsy [4]. Axillary reverse mapping (ARM) is a technique developed based on the premise that upper-extremity lymphedema may result from disruption of lymphatic channels draining the arm during axillary surgery. The lymphatic drainage of the upper extremity is anatomically interconnected with the axillary lymphatic system, including the lateral or brachial lymphatic group located inferior to the axillary vein. ARM aims to distinguish lymphatic channels and lymph nodes draining the upper extremity from those draining the breast during axillary surgery, thereby facilitating preservation of the lymphatic drainage of the arm [5].

In ARM, lymphatic drainage from the upper extremity is mapped using a blue dye, allowing identification of arm-draining lymphatic channels and lymph nodes during ALND. By keeping these tissues intact, the likelihood of postoperative lymphedema may be decreased by preserving lymphatic flow from the upper extremity. However, the anatomical relationship between ARM-identified lymph nodes and the lymph nodes routinely removed during ALND, as well as the feasibility of preserving arm-draining lymphatic structures without compromising oncological clearance, remains an area of clinical interest [6-9].

The present study aimed to evaluate the anatomical relationship of ARM-identified lymphatic structures to the axillary dissection field, compare lymph node yield between patients in whom ARM structures could and could not be preserved, assess postoperative lymphatic drainage outcomes, and evaluate the feasibility of identifying and selectively preserving arm-draining lymphatic structures during axillary lymph node dissection. The study also evaluated the association between ARM preservation and postoperative upper-extremity lymphedema. Because group allocation was based on intraoperative anatomical findings rather than random assignment, between-group comparisons were interpreted as observational associations rather than evidence of a causal treatment effect.

## Materials and methods

Study design and setting

This prospective comparative study was conducted at the MNJ Institute of Oncology and Regional Cancer Centre, Hyderabad, Telangana, India, over two years from May 2021 to May 2023. The study included 100 adult female patients with carcinoma of the breast who underwent modified radical mastectomy (MRM) with upfront ALND. All eligible patients were prospectively enrolled and evaluated according to a predefined study protocol. The study obtained ethical approval from the Institutional Ethics Committee (IEC), Osmania Medical College, via reference number ECR/300/Inst/AP/2013/RR-20.

Study population

Female patients aged > 18 years with histologically diagnosed carcinoma of the breast who required upfront ALND were considered eligible for the study. Patients who provided informed consent to participate and completed the designated study proforma were enrolled.

Eligibility criteria

Patients aged > 18 years with carcinoma of the breast requiring upfront ALND were included. Patients with an allergy to methylene blue dye, previous surgery involving the ipsilateral axilla, previous axillary radiotherapy, previous chemotherapy, a history of primary lymphedema, extensive axillary lymph node metastasis, pregnancy, or breastfeeding were excluded.

Axillary reverse mapping technique

2.5 mL of methylene blue dye was subcutaneously injected into the upper inner aspect of the ipsilateral arm along the medial intramuscular groove after the simple mastectomy was completed, 5-10 minutes before ALND. This area was chosen due to the tattoo's less noticeable placement and comparatively quick lymphatic drainage. After the injection, the arm was raised for around five minutes to promote lymphatic drainage, and the injection site was gently rubbed. During axillary dissection, the blue-stained lymphatic channels and lymph nodes draining the upper extremity were identified, and their anatomical relationship with the axillary dissection field was documented.

ARM mapping did not replace or intentionally limit the planned ALND. The decision to preserve an ARM-stained lymphatic channel or lymph node was based on its anatomical location outside the required axillary dissection field. When an ARM-stained structure was located within the required dissection field, it was sacrificed as part of the axillary dissection. Thus, preservation of ARM structures was not intended to result in the omission of lymphatic tissue that would have required removal for the planned axillary dissection.

Study groups

Based on the intraoperative location of the ARM-identified lymphatic channels and lymph nodes in relation to the axillary dissection field, patients were categorized into two groups. Group A comprised patients in whom ARM lymphatic channels and lymph nodes were located outside the required axillary dissection field and could therefore be identified and preserved. Group B comprised patients in whom ARM lymphatic channels and/or lymph nodes were located within the required axillary dissection field and therefore could not be preserved without including them in the axillary dissection. These ARM structures were consequently sacrificed during the axillary dissection. Thus, group allocation was determined by intraoperative anatomical findings and not by random assignment.

Assessment of postoperative lymphedema

Postoperative upper-extremity lymphedema was assessed using the methodology described in previous studies of ARM [10]. Arm circumference was measured at a standardized point 10 cm proximal to the medial epicondyle of the upper extremity. Preoperative arm circumference measurements were used as the baseline, and postoperative assessment was performed at six months following surgery. The circumference of the operated arm was compared with the preoperative measurement and with the contralateral upper extremity. Postoperative lymphedema was defined as an increase in arm circumference of more than 2 cm during the follow-up period. All patients were reassessed at six months, and the presence or absence of lymphedema was recorded.

Outcome measures

The primary outcome was the development of postoperative upper-extremity lymphedema. Secondary outcomes included lymphocele formation, postoperative drain output, duration of drainage until drain removal, total axillary lymph node yield, ARM-identified lymph node yield, and the presence of metastatic disease in ARM-stained lymph nodes. Operative laterality was also recorded.

Data collection

Demographic, clinical, intraoperative, and postoperative data were prospectively collected using a predefined study proforma. Patient age and operative laterality were recorded. Intraoperative findings, including identification and location of ARM lymphatic channels and lymph nodes, were documented. The total axillary lymph node yield, ARM lymph node yield, and presence of metastatic disease in ARM-stained lymph nodes were recorded. Postoperative drain output, day of drain removal, lymphocele formation, and postoperative lymphedema were assessed and documented during follow-up.

Data analysis

The data were first imported into Microsoft Excel and then evaluated using IBM SPSS Statistics, version 27.0 (IBM Corp., Armonk, NY). Continuous variables were displayed as mean ± standard deviation (SD), while categorical data were displayed as frequencies and percentages. Age, total lymph node yield, ARM lymph node yield, postoperative drain output, and drain-removal day were among the continuous variables that were compared between the two groups using the independent-samples t-test. Categorical factors, including lymphocele development, postoperative lymphedema, and operation laterality, were compared using the chi-square test. In all two-sided statistical tests, a p-value of less than 0.05 was considered statistically significant.

## Results

There were 100 patients in the study, with 50 in each group. The mean age of Group B was 51.28 ± 12.584 years, while that of Group A was 50.08 ± 11.866 years. The two groups did not differ significantly (t = −0.491, p = 0.625). Regarding the operating side, 31 (62.0%) and 25 (50.0%) patients, respectively, underwent right-sided surgery, while 25 (50.0%) patients in Group B and 19 (38.0%) patients in Group A underwent left-sided surgery. The distribution of the operative side was not significantly different between the groups (χ² = 1.461, p = 0.227), indicating comparable baseline demographic and operative laterality characteristics (Table 1).

**Table 1:** Demographic characteristics and operative side of the study population SD: standard deviation

Variable	Group A (n = 50)	Group B (n = 50)	Test statistic	P-value
Age, years, mean ± SD	50.08 ± 11.866	51.28 ± 12.584	t = −0.491	0.625
Operative side, n (%)			χ² = 1.461	0.227
Left	19 (38%)	25 (50%)		
Right	31 (62%)	25 (50%)		

Postoperative lymphedema occurred in six (12.0%) patients in Group A compared with nine (18.0%) patients in Group B. Although the incidence was numerically lower in Group A, the difference was not statistically significant (χ² = 0.706, p = 0.401). Similarly, there was no statistically significant difference between the groups in terms of lymphocele development (χ² = 1.042, p = 0.307), with lymphocele development occurring in one (2.0%) patient in Group A and three (6.0%) patients in Group B (Table 2).

**Table 2:** Postoperative complications by study group

Postoperative outcome	Group A (n = 50)	Group B (n = 50)	Chi-square test	P-value
Lymphedema, n (%)			χ² = 0.706	0.401
Yes	6 (12%)	9 (18%)		
No	44 (88%)	41 (82%)		
Lymphocele, n (%)			χ² = 1.042	0.307
Yes	1 (2%)	3 (6%)		
No	49 (98%)	47 (94%)		

Group A's mean total lymph node yield was 9.84 ± 3.177, while that of Group B was 10.96 ± 3.398. There was no statistically significant difference between the two groups (t = −1.702, p = 0.092). In contrast, Group B had a considerably greater ARM lymph node yield than Group A (1.38 ± 0.53 vs. 0 ± 0; p = 0.001). With a mean yield of 0 ± 0 in Group A and 0.04 ± 0.198 in Group B, metastatic ARM-stained lymph nodes were rare; however, this difference was not statistically significant (t = 1.428, p = 0.155) (Table 3).

**Table 3:** Lymph node yield and ARM findings by study group ^*^Statistically significant A p-value less than 0.05 was considered significant ARM: axillary reverse mapping; SD: standard deviation

Variable	Group A (n = 50)	Group B (n = 50)	Student's t-test value	P-value
Total lymph node yield, mean ± SD	9.84 ± 3.177	10.96 ± 3.398	−1.702	0.092
ARM lymph node yield, mean ± SD	0 ± 0	1.38 ± 0.53	18.4	0.001^*^
Metastatic ARM-stained lymph nodes, mean ± SD	0 ± 0	0.04 ± 0.198	1.428	0.155

Group A's mean postoperative drain output was 337 ± 183.181, whereas that of Group B was 353 ± 199.055. There was no statistically significant difference between the two groups (t = −0.418, p = 0.676). Group A's mean drain-removal day was 8.92 ± 1.759, whereas that of Group B was 9.90 ± 1.810. The results presented in the table indicated that this difference was statistically significant (t = 2.74, p = 0.007) (Table 4).

**Table 4:** Postoperative drain outcomes by study group ^*^Statistically significant A p-value less than 0.05 was considered significant SD: standard deviation

Variable	Group A (n = 50)	Group B (n = 50)	Student's t-test value	P-value
Drain output, mean ± SD	337 ± 183.181	353 ± 199.055	−0.418	0.676
Drain removal day, mean ± SD	8.92 ± 1.759	9.90 ± 1.810	2.74	0.007^*^

## Discussion

ALND remains an important component of surgical management in selected patients with breast cancer; however, disruption of lymphatic drainage from the upper extremity during ALND can result in postoperative lymphedema. ARM was developed to identify lymphatic channels and lymph nodes draining the arm, allowing their preservation when oncologically appropriate [7,8]. In the present study, ARM preservation was feasible during MRM. Patients in whom ARM structures could be preserved had a numerically lower incidence of postoperative lymphedema; however, this difference was not statistically significant, and the non-random, anatomy-based group allocation precludes interpreting this finding as a causal treatment effect.

The two study groups were comparable with respect to age and operative laterality, supporting their overall comparability. The mean total lymph-node yield was 9.84 ± 3.177 in Group A and 10.96 ± 3.398 in Group B, with no statistically significant difference (p = 0.092). This finding is important because preservation of ARM structures should not compromise the adequacy of axillary clearance. The significantly greater ARM lymph-node yield in Group B compared with Group A (1.38 ± 0.53 vs. 0; p = 0.001) reflected the predefined difference in the management of ARM structures between the groups.

Postoperative lymphocele formation was uncommon and did not differ significantly between groups (2.0% vs. 6.0%; p = 0.307). Similarly, mean drain output was comparable between Group A and Group B (337 ± 183.181 vs. 353 ± 199.055; p = 0.676). In contrast, the mean drain-removal day was significantly lower in Group A than in Group B (8.92 ± 1.759 vs. 9.90 ± 1.810 days; p = 0.007). This finding indicates an association between group membership and earlier drain removal; however, because group allocation was based on intraoperative anatomical findings rather than random assignment, the difference should not be interpreted as a causal effect of ARM preservation. Early studies demonstrated the feasibility of ARM and provided the anatomical basis for selective preservation of arm lymphatic structures during axillary surgery [7,8,11].

Kang et al. [12] and Ponzone et al. [13] further highlighted the potential lymphatic benefit of ARM while emphasizing the possibility of crossover between breast and arm lymphatic drainage. Noguchi et al. [14] demonstrated the feasibility of fluorescence-guided ARM for identifying arm-draining lymphatic structures, while Boneti et al. [15] suggested that mapping and preservation of arm lymphatics may help reduce postoperative lymphedema.

More recent comparative evidence has generally demonstrated a stronger protective effect. Faisal et al. [10], in a randomized controlled trial, reported lymphedema in 4.2% of patients undergoing ARM-guided preservation compared with 16.7% following conventional ALND. Gennaro et al. [16] similarly demonstrated a lower incidence of breast cancer-related lymphedema following reverse lymphatic mapping with selective axillary dissection compared with standard axillary treatment. A meta-analysis of five randomized controlled trials involving 1,659 patients reported lymphedema rates of 4.71% with ARM compared with 18.79% following conventional ALND [17]. Wijaya et al. [18], in a systematic review and meta-analysis involving 4,954 patients, also found that ARM preservation significantly reduced upper-extremity lymphedema compared with ARM resection. Although the present study did not demonstrate statistical significance, the observed reduction from 18.0% to 12.0% was directionally consistent with these findings. The relatively small sample size and differences in follow-up duration and the methods used for lymphedema assessment may partly explain the absence of statistical significance.

The oncological safety of ARM preservation remains an important consideration because arm and breast lymphatic drainage are not completely separate. In the present study, metastatic ARM-stained lymph nodes were uncommon, with no statistically significant difference between the groups (p = 0.155). Previous studies have reported variable rates of ARM-node metastasis, particularly in patients with extensive axillary disease [10-12]. Ponzone et al. demonstrated an association between ARM-node involvement and extensive axillary disease, emphasizing that preservation should not be indiscriminate [12]. Therefore, ARM structures that appear suspicious or are involved by metastatic disease should be excised to maintain oncological safety.

Limitations

This study has several limitations, including its single-center design and somewhat small sample size. Lymphedema is also influenced by factors such as radiotherapy, body mass index, extent of axillary disease, number of nodes removed, and the duration and method of follow-up. An important limitation is the non-random, anatomy-based allocation of patients into the two study groups. Because group assignment depended on the intraoperative anatomical relationship of ARM structures to the axillary dissection field, unmeasured anatomical or oncological characteristics may have differed between groups and could have influenced postoperative outcomes. Therefore, the observed differences between groups, including the incidence of lymphedema, should be interpreted as observational associations rather than causal effects of ARM preservation.

Larger randomized or appropriately controlled prospective studies are required to establish the independent effect of ARM preservation on postoperative lymphedema. Overall, the present findings support ARM as a feasible lymphatic-preservation technique that may reduce postoperative lymphedema without compromising axillary nodal yield. However, given the possibility of ARM-node metastasis, preservation should remain selective and guided by oncological considerations.

## Conclusions

ARM is a feasible technique for identifying and selectively preserving upper-extremity lymphatic structures during MRM with ALND. In the present study, ARM preservation was not associated with a statistically significant difference in total axillary lymph node yield. Postoperative lymphedema occurred less frequently in the ARM-preservation group than in the group in which ARM structures were sacrificed; however, this difference was not statistically significant. Given the non-random, anatomy-based allocation of patients, this finding should be interpreted as an observational association rather than evidence of a causal preventive effect. A significant difference was observed in the duration of drain placement between the groups, whereas no significant differences were observed in lymphocele formation or postoperative drain output. The low frequency of metastatic ARM-stained lymph nodes supports selective preservation in appropriately selected patients; however, suspicious or oncologically involved ARM lymphatic structures should be excised to maintain oncological safety. Larger prospective multicenter studies with standardized lymphedema assessment and longer follow-up are warranted to further evaluate the potential role of ARM in reducing postoperative lymphedema and to establish its long-term oncological safety.
